# Association between blood heavy metal exposure levels and risk of metabolic dysfunction associated fatty liver disease in adults: 2015–2020 NHANES large cross-sectional study

**DOI:** 10.3389/fpubh.2024.1280163

**Published:** 2024-02-16

**Authors:** Song Tang, Simin Luo, Zhendong Wu, Jiandong Su

**Affiliations:** ^1^Department of Gastrointestinal Surgery, The First Affiliated Hospital of Guangdong Pharmaceutical University, Guangzhou, China; ^2^Breast Tumor Center, Sun Yat-sen Memorial Hospital, Sun Yat-sen University, Guangzhou, China; ^3^Department of Gastroenterology, Dongguan Songshan Lake Tungwah Hospital, Dongguan, China

**Keywords:** MAFLD, heavy metal, NHANES, manganese, selenium

## Abstract

**Background:**

The relationships between heavy metals and fatty liver, especially the threshold values, have not been fully elucidated. The objective of this research was to further investigate the correlation between blood heavy metal exposures and the risk of Metabolic dysfunction Associated Fatty Liver Disease (MAFLD) in adults.

**Methods:**

Laboratory data on blood metal exposure levels were obtained from National Health and Nutrition Examination Survey (NHANES) data for the period 2015 to 2020 for a cross-sectional study in adults. Associations between blood levels of common heavy metals and the risk of MAFLD in adults were analyzed using multifactorial logistic regression and ranked for heavy metal importance using a random forest model. Finally, thresholds for important heavy metals were calculated using piecewise linear regression model.

**Results:**

In a multifactorial logistic regression model, we found that elevated levels of selenium (Se) and manganese (Mn) blood exposure were strongly associated with the risk of MAFLD in adults. The random forest model importance ranking also found that Se and Mn blood exposure levels were in the top two positions of importance for the risk of disease in adults. The restricted cubic spline suggested a non-linear relationship between Se and Mn blood exposure and adult risk of disease. The OR (95% CI) for MAFLD prevalence was 3.936 (2.631–5.887) for every 1 unit increase in Log Mn until serum Mn levels rose to the turning point (Log Mn = 1.10, Mn = 12.61 μg/L). This correlation was not significant (*p* > 0.05) after serum Mn levels rose to the turning point. A similar phenomenon was observed for serum Se levels, with a turning point of (Log Se = 2.30, Se = 199.55 μg/L).

**Conclusion:**

Blood heavy metals, especially Se and Mn, are significantly associated with MAFLD in adults. They have a non-linear relationship with a clear threshold.

## Introduction

MAFLD, is the most common cause of chronic liver disease worldwide (1). Originally referred to as non-alcoholic fatty liver disease (NAFLD), but proposed to be redefined by an international expert group in 2020 (2). MAFLD is a condition of liver fat accumulation with metabolic dysfunction in the form of overweight or obesity and insulin resistance (3). MAFLD is closely related to diabetes, chronic kidney disease, cardiovascular disease (4) and is also prevalent in patients with hepatocellular carcinoma (5, 6), posing a significant potential economic burden to society.

The relationship between heavy metals and liver diseases has been confirmed in previous studies. One study showed that liver function was significantly correlated with blood heavy metals, and different heavy metals had different effects on various indicators of liver function (7). Study has also shown a positive association between blood Se and NAFLD (8). In addition, blood Mn and Se are associated with hepatic steatosis and fibrosis (9). However, previous studies have mostly focused on the effects of heavy metals on the liver, lacking of description of the thresholds for the relationship between heavy metal exposure levels and the risk of developing MAFLD in adults. Given the importance of thresholds in describing relationship, the relationship between heavy metal exposures and the risk of MAFLD in adults must be further explored.

This study retrieved the data of the NHANES from 2015 to 2020, used multi-factor logistics regression to analyze the association between blood levels of common heavy metals and the risk of MAFLD in adults, and ranked the importance of heavy metals by random forest model. Furthermore, the piecewise linear regression model was used to calculate the threshold values of the two most significantly correlated heavy metals, which is important for understanding the relationship between exposure to heavy metals and MAFLD in adults.

## Methods

### Study population

The NHANES is a cross-sectional survey administered by the National Center for Health Statistics (NCHS) and the Centers for Disease Control and Prevention with data from the civilian population of the United States and is nationally representative. The data collection protocol was approved by the NCHS Ethics Review Committee and all survey participants provided informed consent prior to being interviewed and examined. Because laboratory data on blood metal exposure levels were complete for the period 2015 to 2020 compared to other years, the NHANES public data file for that cycle was used to construct the dataset for this study, and the study population consisted of all NHANES respondents.

### Exposure

The exposure variable in this study was blood heavy metal levels. Blood levels of 10 different metals [plumbum (Pb), hydrargyrum (Hg), cadmium (Cd), Mn, Se, chromium (Cr), cobalt (Co), inorganic hydrargyrum (InHg), methyl hydrargyrum (MeHg) and ethyl hydrargyrum (EtHg)] were obtained by direct extraction of participant laboratory data. The test method is described in detail in the NHANES database^1^ and focuses on the direct measurement of heavy metals in whole blood samples using mass spectrometry after a simple dilution sample preparation procedure.

### Outcome

The primary outcome of this study was the presence or absence of MAFLD, which was defined as the combination of steatosis, irrespective of the gradation, and metabolic dysfunction. This was characterized by either overweight (Body mass index (BMI) ≥25 kg/m^2^), type 2 diabetes mellitus defined as antidiabetic drug use, fasting plasma glucose ≥7.0 mmol/L, HbA1c > 6.4% or based on oral glucose tolerance test (OGTT), or a combination of at least two of the following metabolic abnormalities: (1) waist circumference > 102 cm for male and > 88 cm for female; (2) blood pressure ≥ 130/85 mmHg or antihypertensive drug use; (3) plasma triglycerides ≥1.70 mmol/L or lipid-lowering drug treatment; (4) high-density lipoprotein cholesterol (HDL-C) <1.0 mmol/L for men and < 1.3 mmol/L for women or lipid-lowering drug treatment; (5) prediabetes defined as fasting plasma glucose 5.6–6.9 mmol/L, HbA1c 5.7–6.4% or matching OGTT; (6) homeostatic model assessment of insulin resistance (HOMA-IR) of ≥2.5; (7) or C-reactive protein (CRP) level > 2 mg/L (10). Those who met the diagnostic criteria were identified as MAFLD patients, those who did not were identified as controls, and those with missing diagnosis-related data were removed.

### Baseline data on the study population

Demographic characteristics, such as age, gender and ethnicity were included. Social factors, including education level, marital status, ratio of household income to poverty level and health insurance coverage were included. Data on everyday health-related behaviors were collected for smoking and alcohol consumption. Variables related to medical comorbidities, such as liver and kidney function, BMI, diabetes, hypertension and cancer, are collected.

### Data analysis

The quantitative data met a normal distribution by selecting a t-test or analysis of variance (ANOVA), or a rank sum test if they did not meet a normal distribution. Data on categorical variables were analyzed for differences in baseline characteristics and blood heavy metal levels between outcome groups using the *χ*^2^ test. We used Spearman correlation matrices to identify correlations for the 10 heavy metals. Subsequently, multifactorial logistic regression models were used to identify exposure factors associated with the screening outcome variables for the preliminary analysis. A random forest (RF) model was used to determine the importance of the variables. Feature importance was assessed based on the out-of-bag (OOB) error rate, reflecting the level of contribution of each variable when classifying the metabolic dysfunction-related fatty liver with the control population.

To explore the non-linear relationship between blood heavy metal concentrations and the risk of MAFLD, as well as the threshold effect, serum heavy metal concentrations were transformed on a Log10 scale. We used the Restrictive Cubic Spline (RCS) function to analyze the odds ratio (OR) relationship between blood heavy metal concentrations and the risk of MAFLD, as serum heavy metal concentrations showed a skewed distribution. Additionally, a piecewise linear regression model was applied to examine the threshold effect of serum heavy metal concentrations on the risk of MAFLD using a smoothing function. Threshold levels (i.e., turning points) were determined by iterative trials involving the selection of turning points along predefined intervals, followed by the selection of turning points that gave the maximum model likelihood.

All data analyzes were conducted using R.3.5.2/R4.2.2.^2^ Sample sizes were based on available data and no ex ante sample size calculations were performed. Significance was tested for all descriptive analyzes by two-sided tests at the *p* < 0.05 level of significance.

## Results

### Baseline characteristics of the study population

**Figure 1:**
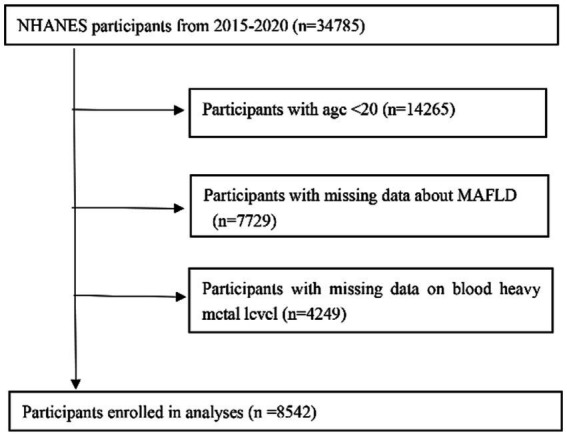
Flow diagram of the screening and selection process.

Survey data were collected from 34,785 participants. After excluding those lacking MAFLD, heavy metal blood level data (with the exceptions mentioned above), 8,542 adult participants (age ≥ 20 years) were included in the analysis (Figure 1). The distribution of baseline characteristics stratified by outcome is shown in Table 1. In the preliminary analysis, the MAFLD group participants, 53.14% male, were significantly higher than the Non-MAFLD group, in addition the age distribution of participants in the MAFLD group was not significantly different from the Non-MAFLD group. In terms of social factors, the proportion of Married or living with partner was higher in the MAFLD group than in the Non-MAFLD group, and there was no significant difference in the education level of the two groups. For comorbidities, BMI was significantly higher in the MAFLD group than in the Non-MAFLD group (33.05 ± 6.84 vs. 26.97 ± 5.56, *p* < 0.001). estimated glomerular filtration rate (eGFR), alanine transaminase(ALT) and aspartate transaminase (AST) were in the normal range in both groups, and HbA1c was in the normal range in the Non-MAFLD group, while HbA1c was above the normal range in the MAFLD group. The MAFLD group had a higher proportion of combined hypertension and diabetes than Non-MAFLD, and the difference was statistically significant (*p* < 0.05).

**Table 1:** Characteristics of participants enrolled in study.

Characteristic	Non-MAFLD (*N* = 4,172)	MAFLD (*N* = 4,370)	*p*-value
Age (y)	60 (50, 69)	60 (51, 68)	0.377
Male sex	1899 (45.52)	2,322 (53.14)	<0.001
Race			<0.001
Hispanic	756 (18.12)	1,103 (25.24)	
Non-Hispanic white	1,441 (34.54)	1,644 (37.62)	
Non-Hispanic black	1,182 (28.33)	936 (21.42)	
Other	793 (19.01)	687 (15.72)	
Education beyond high school	3,322 (79.72)	3,435 (78.82)	0.305
Marital status			0.011
Never married	135 (8.63)	147 (8.65)	
Married or living with partner	931 (59.49)	1,090 (64.16)	
Divorced, separated, or widowed	499 (31.88)	462 (27.19)	
BMI (kg/m2)	26.1 (23.3, 29.7)	31.7 (28.1, 36.4)	<0.001
eGFR, ml/min per 1.73 m2	86.93 (71.87, 100.04)	88.48 (72.52, 100.95)	0.037
ALT (U/L)	16 (12, 22)	20 (15, 28)	<0.001
AST (U/L)	19 (16,23)	20 (16,25)	0.001
HbA1c	5.6 (5.4, 5.9)	5.9 (5.5, 6.6)	<0.001
Alcohol user	2,535 (86.28)	2,725 (87.62)	0.123
Smoker	1855 (44.48)	1995 (45.66)	0.274
Hypertension	1916 (45.93)	2,644 (60.50)	<0.001
Cancer	531 (12.73)	600 (13.74)	0.172
Diabetes	709 (16.99)	1705 (39.02)	<0.001

All values are displayed as *n* (%) or median and IQR. *χ*^2^ analysis is used to test significance between groups for categorical variables.BMI, body mass index; eGFR, estimated glomerular filtration rate; HbA1c, glycosylated Hemoglobin, type A1C; ALT, alanine aminotransferase; AST, aspartate aminotransferase.

### Blood levels of heavy metal exposure in the study population

In the preliminary analysis, the 10 heavy metal blood exposure levels were skewed data. Comparing the participants in the Non-MAFLD group, there were differences in the distribution of heavy metal blood levels in Pb, Hg, Mn, Se, MeHg, InHg, Cd and Co among the participants in the MAFLD group, with Mn and Se levels being high and the differences being statistically significant (*p* < 0.05) (Table 2).

**Table 2:** Level of blood heavy metals in Non-MAFLD and MAFLD subjects.

Characteristic	Non-MAFLD (*N* = 4,172)	MAFLD (*N* = 4,370)	*p*-value
Co (μg/L)	0.15 [0.12, 0.19]	0.15 [0.12, 0.18]	<0.001
Cr (μg/L)	0.29 [0.29, 0.29]	0.29 [0.29, 0.29]	0.015
EtHg (μg/L)	0.05 [0.05, 0.05]	0.05 [0.05, 0.05]	0.835
MeHg (μg/L)	0.59 [0.18, 1.42]	0.51 [0.18, 1.25]	<0.001
InHg (μg/L)	0.15 [0.15, 0.23]	0.15 [0.15, 0.22]	0.008
Se (μg/L)	183.52 [169.09, 201.47]	188.11 [172.50, 204.48]	<0.001
Mn (μg/L)	8.92 [7.00, 11.21]	9.18 [7.500, 11.38]	<0.001
Cd (μg/L)	0.38 [0.23, 0.66]	0.30 [0.190, 0.50]	<0.001
Hg (μg/L)	0.79 [0.30, 1.70]	0.69 [0.360, 1.50]	<0.001
Pb (μg/L)	1.17 [0.77, 1.77]	0.99 [0.67, 1.51]	<0.001

All values are displayed as Median (Quartile1-Quartile 3).

### Influence of blood heavy metal exposure levels on the risk of MAFLD

**Figure 2:**
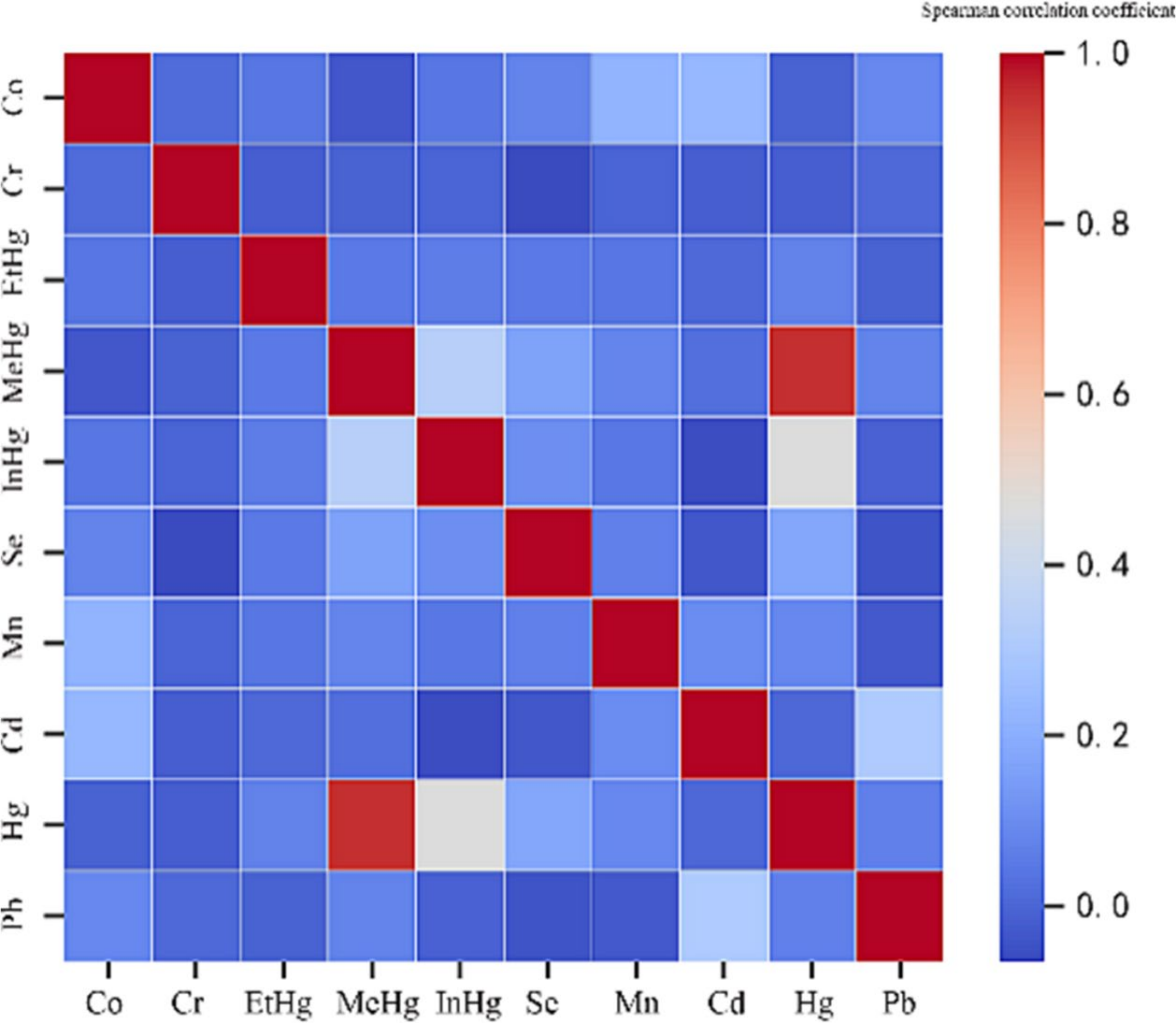
Heat maps of correlations between 10 environmental heavy metals.

Firstly, Spearman’s correlation matrix analysis (Figure 2) was performed for the correlation of 10 environmental heavy metals. There was a significant correlation between MeHg and Hg (correlation value of 0.951, *p*﹤0.001). Therefore, MeHg should be eliminated in the next Poisson review. Exploratory factor analysis confirmed highly correlated covariates, with the other nine metals having limited correlation with each other.

**Figure 3:**
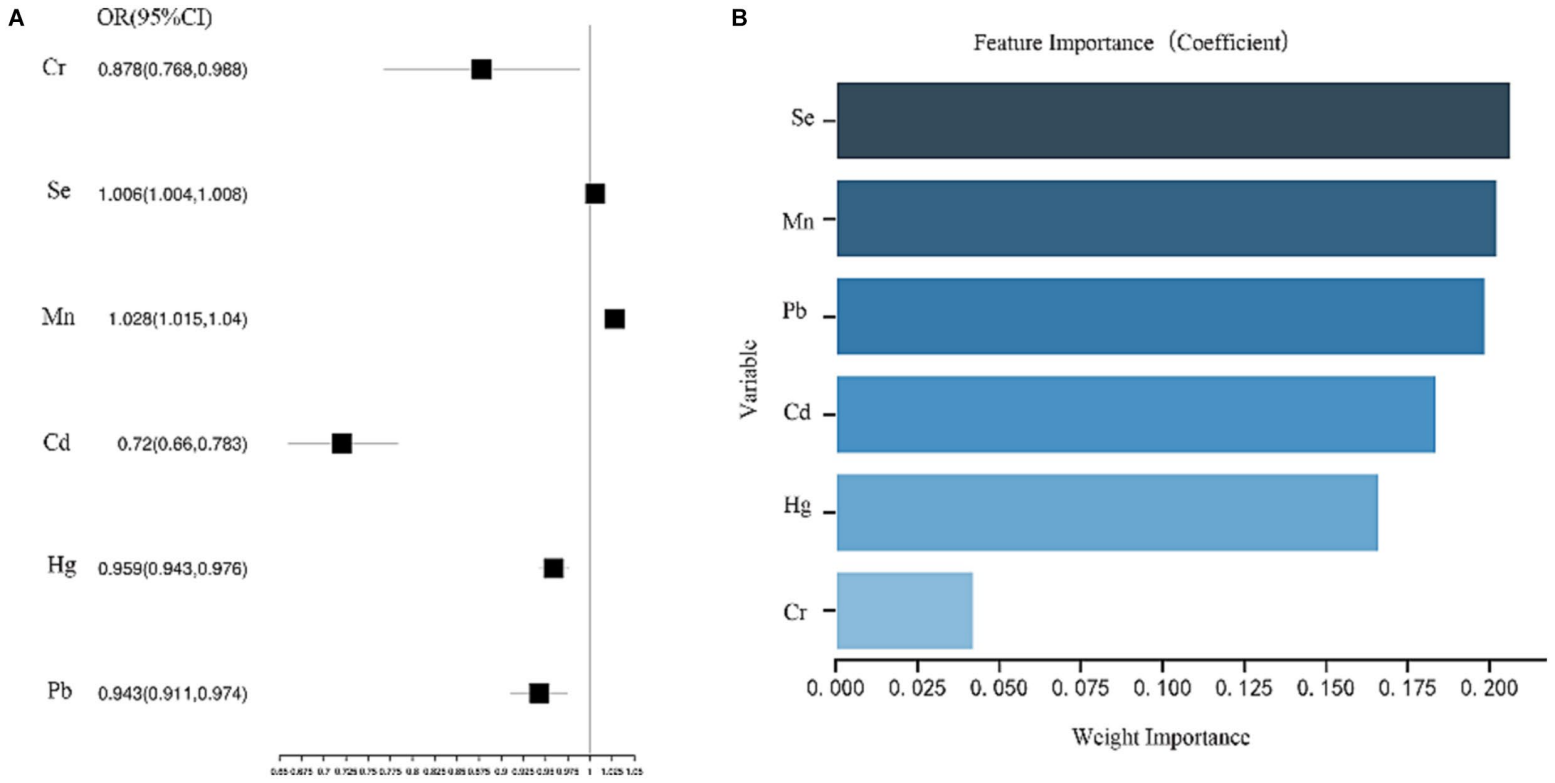
**(A)** Analysis results of multi-factor logistics regression model. **(B)** The order of importance of variables.

Analysis using a multifactorial logistic regression model found that high blood Mn and Se exposure levels were associated with an increased risk of disease in patients in the MAFLD group compared to the Non-MAFLD group [OR (95% CI): Mn 1.028 (1.015–1.04); Se 1.006 (1.004–1.008)] (Figure 3A). The random forest model determined the importance of the variables, with the top 2 rankings of characteristic importance Se and Mn, which matched the multi-factor logistic regression model (Figure 3B).

### Threshold effect of blood Mn and Se exposure levels on the risk of MAFLD prevalence

**Figure 4:**
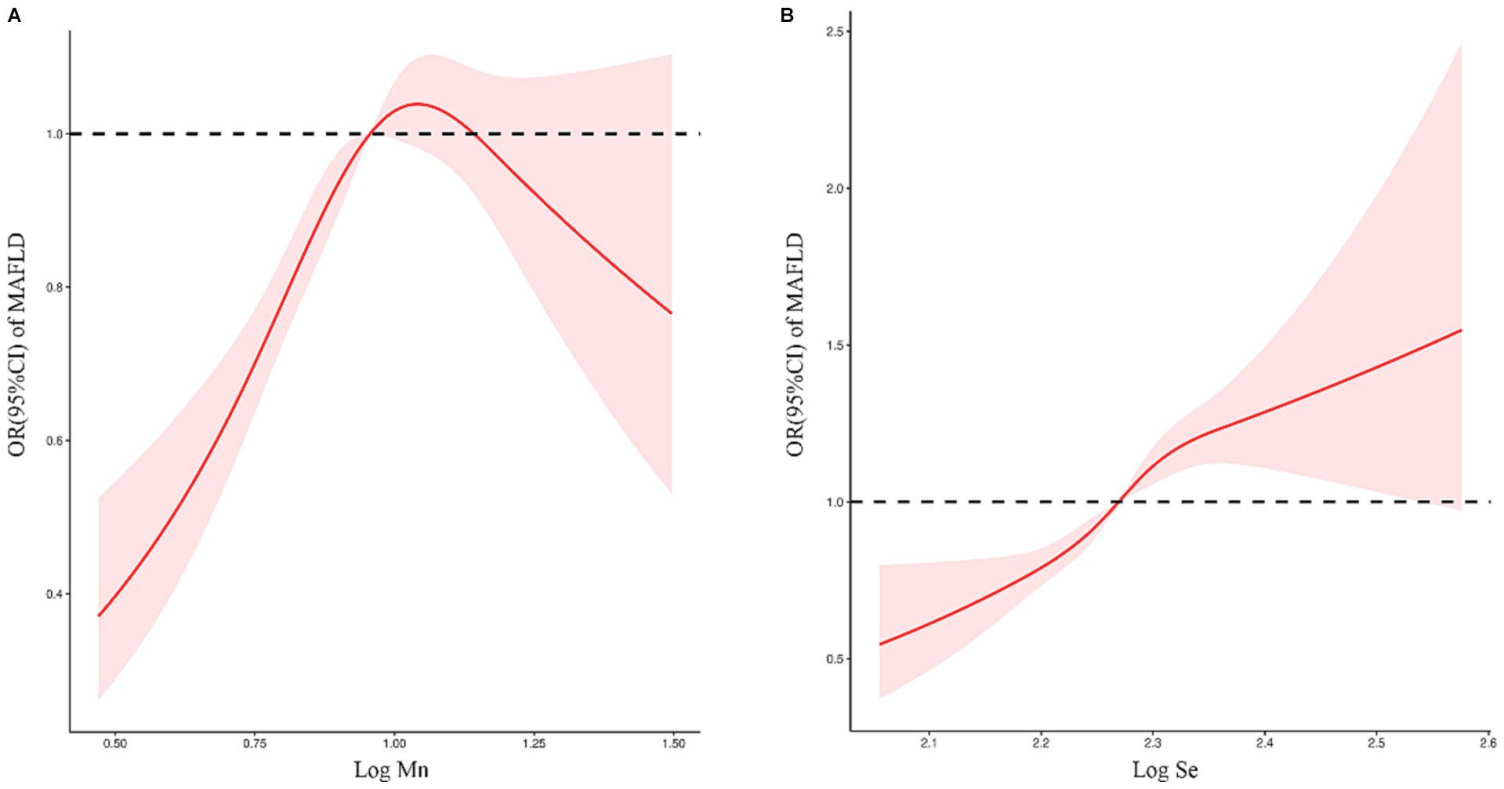
**(A)** Relationship between serum Mn exposure level and risk of MAFLD. **(B)** Relationship between serum Se exposure level and risk of MAFLD.

The RCS plot (Figure 4) shows a non-linear relationship between serum Mn and Se exposure levels and the risk of MAFLD. The OR (95% CI) for MAFLD prevalence (Table 3) was 3.936 (2.631–5.887) for every 1 unit increase in Log Mn until serum Mn levels rose to the turning point (Log Mn = 1.10, Mn = 12.61 μg/L). This correlation was not significant (*p* > 0.05) after serum Mn levels rose to the turning point. A similar phenomenon was observed for serum Se levels, with a turning point of (Log Se = 2.30, Se = 199.55 μg/L).

**Table 3:** Threshold effect analysis of blood heavy metals on MAFLD using piecewise linear regression.

	OR (95% CI)	*p*-value
Se		
Log Se < 2.30	36.438 (10.744–123.576)	<0.001
Log Se ≥ 2.30	5.845 (0.893–38.235)	0.065
Mn		
Log Mn < 1.10	3.936 (2.631–5.887)	<0.001
Log Mn ≥ 1.10	0.458 (0.121–1.726)	0.248

## Discussion

MAFLD, the most common cause of chronic liver disease (1), poses a huge potential economic burden on society. The etiology of MAFLD has not been fully elucidated, with oxidative stress and metabolic imbalance of fatty acids playing a crucial role (11).

Due to the continuous development of human industry, mineral resources have been extensively exploited, leading to severe heavy metal pollution of water and soil, which in turn poses a threat to human health. With the increase in heavy metal pollution, the relationship between heavy metal exposure and metabolic diseases in adults has attracted widespread attention. Studies have shown that the liver is one of the major accumulation organs of heavy metals, especially plumbum, cadmium, nickel and chromium, which accumulate in large quantities in the liver (12). The toxic effects of heavy metals occur mainly through oxidative stress induced by the production of reactive oxygen species (ROS) in cells, which may cause liver damage and lead to the development of fatty liver (13, 14). Previous studies have focused on the effects of heavy metals on the liver, and there is a lack of description of thresholds for the relationship between heavy metal exposures and the risk of developing MAFLD in adults, so more research is needed.

We obtained blood level data for 10 different metals at NHANES from 2015 to 2020 and obtained data on demographic characteristics, social factors, daily health-related behaviors and variables related to medical comorbidities. First, we compared the blood levels of heavy metals between participants in the Non-MAFLD and MAFLD groups and found differences in the distribution of heavy metal blood levels in the MAFLD group for Pb, Hg, Mn, Se, MeHg, InHg, Cd, and Co, with statistically significant differences (*p* < 0.05) for Mn and Se levels. We then analyzed the effect of heavy metal exposure levels on the risk of MAFLD prevalence using a multifactorial logistic regression model and found that high blood Mn and Se exposure levels were associated with an increased risk of prevalence in patients in the MAFLD group. The importance of the variables in the random forest model also indicated that Se and Mn ranked in the top 2, which was consistent with the multi-factor logistic regression model. We then used the RCS model to find a non-linear relationship between serum Mn and Se exposure levels and the risk of MAFLD. Finally, based on this non-linear relationship, we used a piecewise linear regression model to derive a turning point of Log Mn = 1.10 for serum Mn = 12.61 μg/L and Log Se = 2.30 for serum Se = 199.55 μg/L.

Both Mn and Se are controversial and contradictory in influencing the risk of MAFLD. Mn is an essential element for humans and Mn-containing superoxide dismutase (MnSOD) is highly expressed in differentiated organs containing large numbers of mitochondria, such as the heart, liver and kidney, and is the main antioxidant enzyme in mitochondria, playing a key role in the detoxification of superoxide radicals and protecting cells from oxidative stress (15). A clinical study found that there may be sex differences in the association between blood Mn and MAFLD, higher blood Mn may be a potential protective factor for MAFLD in males, but there is no significant association in females (16). The sex difference may be related to the fact that Mn can act as an endocrine disrupter. Study have shown that Mn can increase the level of serum testosterone (17). The association between testosterone and MAFLD differs by sex, with higher testosterone levels decreasing the odds of MAFLD in men and increasing the odds of NAFLD in women (18, 19). In addition, some clinical studies have shown that higher levels of Mn exposure are positively associated with MAFLD and hepatic steatosis degeneration and liver fibrosis (8, 9, 20). However, hepatic steatosis, fibrosis, and hepatocellular carcinoma all occurred more frequently in men than in women (21, 22). This suggests that there is a sex difference in liver disease itself. Perhaps, Mn may have magnified this difference, which requires more study. Mn also has an effect on liver function. In a cross-sectional study of Chinese manganese miners, ALT, AST and direct bilirubin (DBIL) were found to be significantly elevated in workers exposed to high levels of Mn (23). Additional studies have also shown an association between Mn and elevated liver enzyme levels and mortality associated with chronic liver disease (24), with the most significant association between Mn and ALT (7). Animal studies have also shown that Mn exposure can cause liver damage through oxidative stress, mitochondrial damage and thus elevated liver enzymes (25, 26).

We generally believe that the antioxidant effect of Se may attenuate the development of metabolic diseases including MAFLD (27–29). In an animal study, dietary selenium was shown to promote selenoprotein P1 (SEPP1) synthesis and modulate the Kelch-like ECH-associated protein 1 (KEAP1)/NF-E2-related factor 2 (NRF2) pathway to protect against hepatocyte oxidative stress (30). However, several previous clinical studies, including ours, have shown that high levels of selenium are associated with the risk of developing MAFLD, positively correlated with hepatic steatosis (8, 9, 31–33) and positively correlated with changes in liver function, with Se being most significantly associated with changes in ALT (7). We suggest that this may be related to the fact that high dietary selenium intake increases MAFLD risk by modulating dysregulation of insulin biosynthesis and secretion as well as stimulating glucagon secretion, insulin resistance and dyslipidemia (33, 34). It has also been proposed that MAFLD and liver fibrosis are caused by an imbalance in selenium homeostasis rather than by dietary selenium intake (8).

Although our study confirmed the association of Mn and Se with the risk of MAFLD prevalence and also calculated their thresholds precisely, there are still some unavoidable limitations and shortcomings. On the one hand, our study is a retrospective study, based on published data for analysis, and has inherent shortcomings compared to prospective studies. On the other hand, the mechanisms underlying the effects of Mn and Se on MAFLD are not fully understood, and previous studies have had conflicting findings. Therefore, more studies, especially prospective studies and laboratory studies, are needed to further clarify the mechanisms of the effects of Mn and Se on MAFLD.

## Conclusion

Our study further clarified the relationship between blood heavy metal exposure levels and MAFLD in adults. Notably, Se and Mn exhibited the most significant influence on MAFLD. Although higher exposure levels of Se and Mn were linked to an elevated risk of MAFLD, this association demonstrated a non-linear pattern with clear thresholds. Furthermore, the exact mechanism behind the impact of heavy metals on MAFLD remains incompletely understood based on current research, necessitating future prospective and laboratory investigations.

## Data availability statement

The original contributions presented in the study are included in the article/Supplementary material, further inquiries can be directed to the corresponding author.

## Ethics statement

Ethical approval was not required for the studies involving humans because Institutional Review Board approval was not required as the NHANES represents an adequately de-identifed and publicly available dataset. The studies were conducted in accordance with the local legislation and institutional requirements. Written informed consent for participation was not required from the participants or the participants' legal guardians/next of kin in accordance with the national legislation and institutional requirements because the NHANES represents an adequately de-identifed and publicly available dataset.

## Author contributions

ST: Data curation, Formal analysis, Methodology, Project administration, Software, Validation, Visualization, Writing – review & editing. SL: Writing – original draft. ZW: Writing – review & editing. JS: Writing – review & editing.

## Footnotes

^1^ https://www.cdc.gov/nchs/nhanes/index.htm

^2^ http://www.R-project.org
